# German adult population norm values of the short Warwick Edinburgh mental well-being scale (SWEMWBS)

**DOI:** 10.1007/s11136-024-03695-z

**Published:** 2024-06-05

**Authors:** Diana Peitz, Heike Hoelling, Sabine Born, Angelika Schaffrath Rosario, Caroline Cohrdes

**Affiliations:** Department of Epidemiology and Health Monitoring, Robert Koch Institute, Berlin, Germany

**Keywords:** Mental well-being, SWEMWBS, Public mental health, Surveillance, Standardization, Population norms

## Abstract

**Purpose:**

The Warwick-Edinburgh Mental Well-Being Scale represents an internationally established inventory to assess population mental well-being. Particularly the short form (SWEMWBS) is recommended for use in Mental Health Surveillance. In the present study, we present normative data of the SWEMWBS for the German adult population.

**Methods:**

Data from the telephone survey German Health Update (GEDA) in 2022 representative of the German adult population (48.9% women, 18–98 years) was processed to estimate SWEMWBS percentile norm values, T-values, z-values and internationally comparable logit-transformed raw scores for the total sample (*N* = 5,606) as well as stratified by sex, age group and sex with age group combinations.

**Results:**

The average mental well-being was comparable to that of other European countries at *M =* 27.3 (*SD* = 4.0; logit-transformed: *M =* 24.79, SD = 3.73). To provide a benchmark, the cut off for low well-being was set at the 15th percentile (raw score: 23; logit-transformed: 20.73), for high well-being at the 85th percentile (raw score: 32; logit-transformed: 29.31).

**Conclusion:**

The present study provides SWEMWBS norm values for the German adult population. The normative data can be used for national and international comparisons on a population level to initiate, plan and evaluate mental well-being promotion and prevention measures.

**Supplementary Information:**

The online version contains supplementary material available at 10.1007/s11136-024-03695-z.

## Background

Mental health encompasses more than just the absence of mental disorders [1]. Following a dual continua model, mental health is composed of the related but distinct dimensions of psychopathology (i.e., represented by mental disorders) as well as of positive mental health (i.e., represented by mental well-being) [2]. Therefore, both dimensions should be assessed in a national Mental Health Surveillance (MHS) and reporting system such as the German MHS to monitor the mental health of the population comprehensively [3, 4]. Data of both dimensions of the dual continua model provides a reliable basis for evidence-based policy in order to plan, initiate and evaluate measures covering a range of indicators for mental health prevention, promotion, care, and rehabilitation [5].

An internationally established measurement tool designed to assess mental well-being on a population level is the Warwick Edinburgh Mental Well-Being Scale (WEMWBS) [6]. The WEMWBS consists of 14 positively worded items covering *hedonic* (“feeling good”) and *eudaimonic* (“functioning well”) aspects of mental well-being, including positive interpersonal relationships [7, 8]. Originating from the United Kingdom (UK), it showed psychometrically sound properties across several validation studies in diverse countries and populations [6, 9, 10], substantiating its suitability to assess mental well-being in the general population to facilitate public mental health promotion and prevention efforts.

A shortened version (SWEMWBS) with seven items was developed by the authors of the original validation by using Rasch modelling [11]. As a result of the shortening process, the remaining items of the SWEMWBS mainly emphasize psychological functioning (eudaimonic) than subjective feeling states (hedonic). However, since these eudaimonic aspects have been rather neglected in public health so far and can be complemented by other established indicators of psychological well-being (e.g., life satisfaction, happiness), the SWEMWBS represents an asset for the comprehensive depiction of public mental well-being [12, 13]. Due to its brevity and preferable scaling properties, this short version is particularly recommended for use in large-scale population surveys [11]. In studies from the UK as well as from Germany comparing WEMWBS and SWEMWBS, the short version demonstrated a comparable performance in terms of reliability and validity [14].

The scale’s short and long versions are increasingly used by national surveillance systems and thus provide an ideal opportunity for international comparison [14–18]. Another advantage is its great potential for the application and measurement of mental well-being from children aged eleven onwards [17, 18] to enhance comparability across a wide age range. Additionally, the SWEMWBS has been benchmarked against other relevant and well-validated measures such as the PHQ-9, a screening instrument for depressive symptoms [16], expanding the possibilities and flexibility of use for MHS.

Normative data of the SWEMWBS from a general adult population survey have not yet been established for Germany. In this study, we aimed to provide norm values for a population-based German adult total sample as well as stratified by sex, age group and sex with age group combinations offering national and international researchers, practitioners and policy makers a benchmark to compare their results with.

## Materials and methods

### Procedure and participants

Data were gathered in the German Health Update (GEDA) telephone survey from June 2022 to January 2023 (waves 4–10). On behalf of the Robert Koch Institute (RKI), the Berlin-based market and social research institute USUMA GmbH continuously surveys about 1.000 randomly assigned people aged 18 years and older per month from the German-speaking population. In order to approach representativeness as closely as possible, a random sampling procedure including landline and mobile telephone numbers (dual-frame method) was used which guarantees nearly complete coverage of the population in Germany [19, 20]. Furthermore, different selection probabilities of the interviewees were considered in the design weighting (see below). Further details on the sampling procedure, survey methods and population weighting are provided by Allen and colleagues [21].

In total, *N* = 5,606 participants (54.4% women, mean age = 58.3, *SD* = 17.6 age range 18 to 98 years) answered the SWEMWBS and were therefore included in the analyses of this study. Sample characteristics can be obtained from Table 1.

**Table 1:** Sample characteristics

Characteristic	Category	Unweighted *n*	Weighted %	Mental Well-Being		
				Unweighted	Weighted	
				Mean (SD)	Mean (SD)	95% CI
Total		5,606	100	28.0 (3.7)	27.3 (4.0)	(27.1, 27.5)
Sex (at birth)	male	2,559	51.1	28.0 (3.7)	27.3 (4.1)	(27.1, 27.5)
	female	3,047	48.9	28.0 (3.6)	27.3 (3.9)	(27.1, 27.6)
Age group	18–19 years	64	4.2	26.3 (4.0)	25.5 (3.8)	(24.4, 26.6)
	20–24 years	179	6.3	26.3 (3.7)	25.8 (4.2)	(24.8, 26.8)
	25–29 years	219	5.9	26.8 (3.2)	26.5 (3.2)	(25.8, 27.1)
	30–34 years	253	7.6	27.3 (3.6)	27.2 (3.7)	(26.6, 27.8)
	35–39 years	269	8.4	27.2 (3.4)	27.1 (3.6)	(26.4, 27.7)
	40–44 years	346	7.3	27.8 (3.5)	27.3 (4.0)	(26.5, 28.0)
	45–49 years	335	7.2	27.9 (3.4)	27.1 (3.9)	(26.5, 27.8)
	50–54 years	458	7.7	27.9 (3.5)	27.7 (3.5)	(27.3, 28.2)
	55–59 years	668	11.5	27.9 (3.8)	27.5 (4.6)	(26.8, 28.1)
	60–64 years	676	8.1	28.3 (3.7)	27.6 (4.3)	(26.9, 28.2)
	65–69 years	605	7.1	28.8 (3.3)	28.3 (3.7)	(27.8, 28.8)
	70–74 years	501	5.8	28.4 (3.8)	27.8 (3.9)	(27.3, 28.3)
	75–79 years	388	4.7	28.7 (3.5)	28.6 (3.4)	(28.1, 29.1)
	80–84 years	413	4.8	28.1 (3.9)	27.7 (4.1)	(27.1, 28.3)
	85–89 years	167	2.4	28.6 (3.9)	28.4 (4.9)	(26.9, 30.0)
	90 years+	65	1.0	27.2 (4.2)	27.4 (3.8)	(26.2, 28.6)

*Notes*  SD = standard deviation, CI = confidence interval

### Measures

#### Short Warwick-Edinburgh mental well-being scale

Participants completed the German translation of the SWEMWBS [22]. The seven positively worded items were answered on a 5-point scale (1 = ‘none of the time’ to 5 = ‘all of the time’) referring to a two-week period. All item values were summed up to build a total score (range from 7 to 35). Higher scores represent higher mental well-being [6].

#### Sociodemographic characteristics

As part of a larger assessment protocol, participants were asked to indicate their sex at birth (male/ female) and current age in years.

### Statistical analyses

All analyses were carried out with *R* statistics [23] and were weighted based on the actual German population structure in line with the German Federal Statistical Office (state, age, sex; as of December 31, 2020) and the 2018 microcensus (ISCED11 education). The aim of using an adjustment weighting was to calibrate the sample results in the case of nonresponse and thereby enhance the representativity of the sample. In general, the willingness to participate is not the same in all population groups, but differs, for example, according to region, age, sex or level of education. In the adjustment weighting, this different willingness to participate is adjusted for bringing the sample into line with the population distribution of relevant characteristics [21].

To replicate the factorial structure of the SWEMWBS, we performed confirmatory factorial analyses (CFA) with robust weighted-mean squares estimator (WLSM).

Next, standardized *z*-values and *T*-values resulting from latent modelling were estimated for the total sample as well as for each sex (male, female) as a smoothed function of age, which was included in the model in sixteen 5-year Sects. (18–19, 20–24, 25–29, 30–34, 35–39, 40–44, 45–49, 50–54, 55–59, 60–64, 65–69, 70–74, 75–79, 80–84, 85–89, 90–98 years), using the R package *cNorm* [24]. The package cNorm does not require any distributional assumptions and conducts continuous norming by means of smoothed regression models and returns smoothed raw scores corresponding to given standardized scores, which in turn can be expressed as percentiles under the assumption of a normal distribution. addition, For the smoothed raw scores we provide the logit-transformed scores developed by Stewart-Brown and colleagues [11] and used by Ng Fat and colleagues [14] to enable international comparison of mean values and distribution parameters. Cut off-points for low well-being (below the 15th percentile) and high well-being (above the 85th percentile) were set using the percentile distribution approach in accordance with the original instructions and procedures provided by Ng Fat et al. ( [14]; see also https://warwick.ac.uk/fac/sci/med/research/platform/wemwbs/using/howto/).

## Results

Both the one-factorial and three-factorial structure of the SWEMWBS as validated in Peitz et al. [10] could be replicated based on this sample with satisfactory model fit, χ^2^(14) = 538.91, *p* < .001, *CFI* = 0.961, *TLI* = 0.942, *RMSEA* = 0.057, and *SRMR* = 0.049 (one-factor model), respectively χ^2^(11) = 346.22, *p* < .001, *CFI* = 0.977, *TLI* = 0.955, *RMSEA* = 0.049, and *SRMR* = 0.038 (three-factor model).

Weighted and unweighted means and standard deviations for the total sample as well as sex and age groups can be obtained from Table 1. The SWEMWBS weighted overall mean based on the raw score was 27.3 (*SD* = 4.0) and for the logit-transformed score 24.79 (*SD* = 3.73).

**Fig. 1:**
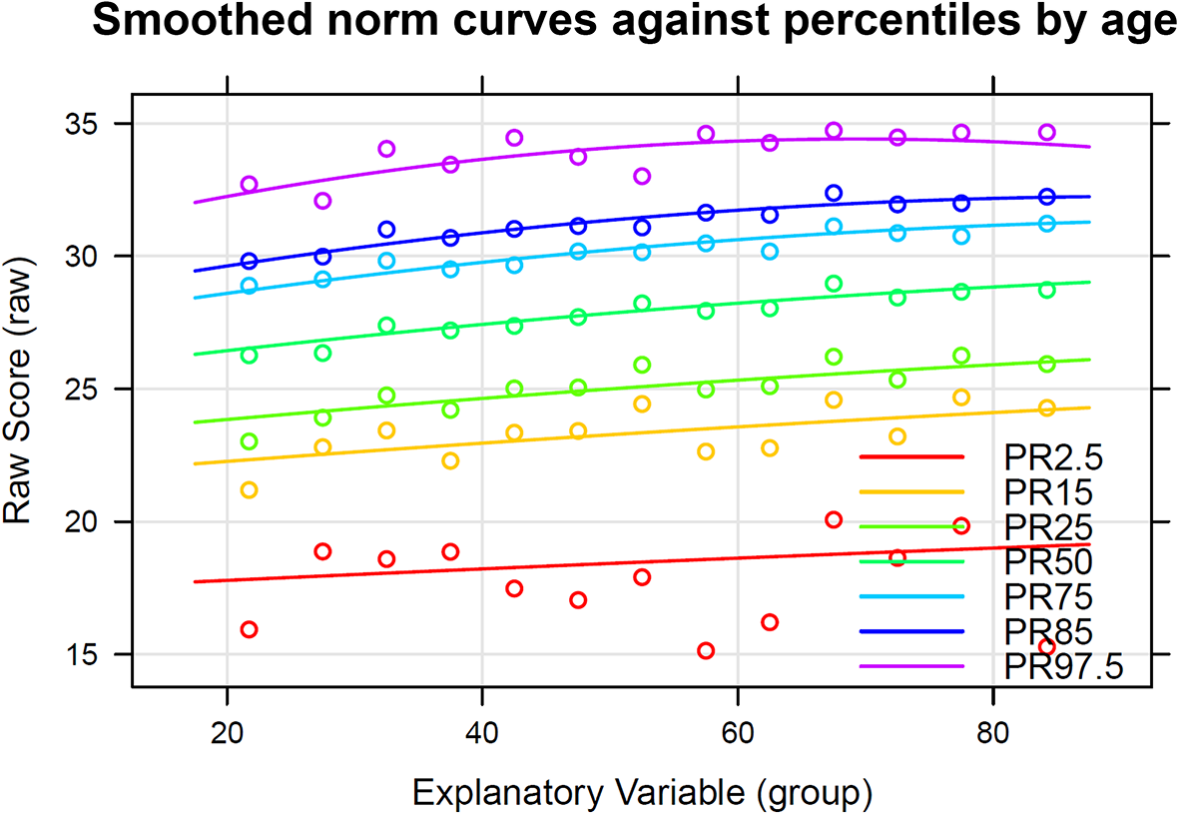
Smoothed norm curves against percentiles by age

Table 2 presents German population norms independent of age, for the total sample as well as for male and female adults separately. The cut-off point for high mental well-being (85th percentile) corresponded to a raw score of 31.4 (logit-transformed: 28.13) and the cut-off point for low well-being (15th percentile) to 23.2 (logit-transformed: 20.73), so that raw scores from 24 to 31 are within the reference range. The predicted smoothed population norms across the age range are shown in Fig. 1 and in the supplementary material in Table S1 (separated by sex in the supplementary material Figures S1 and S2 as well as Table S2). The top age-specific 15% of the smoothed SWEMWBS raw scores ranged from 29.3 to 32.1 and the bottom 15% from 22.1 to 23.9. Figure S1 and Figure S2 suggested an interaction effect between age and sex. Post-hoc regression analyses confirmed a significant interaction term (data not shown), showing lower mental well-being in young women compared to young men.

**Table 2:** SWEMWBS percentiles, z-values, T-values, raw scores and logit-transformed scores, based on *N* = 5,606 German adults in total and stratified by sex

Percentile	T-value	z-value	Total		Male		Female	
			Raw score	Logit-transformed	Raw score	Logit-transformed	Raw score	Logit- transformed
2.5	30.40	-1.96	18.3	17.43	18.0	17.43	18.5	17.98
15	39.64	-1.04	23.2	20.73	23.1	20.73	23.3	20.73
25	43.26	-0.67	24.9	22.35	24.9	22.35	24.9	22.35
50	50	0	27.8	25.03	27.8	25.03	27.7	25.03
75	56.74	0.67	30.2	27.03	30.3	27.03	30.2	27.03
85	60.36	1.04	31.4	28.13	31.5	29.31	31.3	28.13
97.5	69.60	1.96	33.8	32.55	33.9	32.55	33.7	32.55

*Notes* The allocation of logit-transformed values to the (rounded) raw scores was based on Stewart-Brown and colleagues [11]

## Discussion

Mental well-being has become a crucial asset for monitoring and evaluating public mental health. There is accumulating evidence that the SWEMWBS is a sufficient tool for observing population-based trends and cross-national comparison [6, 9, 11, 14]. Thus, in the German MHS, it is planned to continuously assess, interpret, and report mental well-being with the SWEMWBS. Up to now, no representative norm values for Germany were available to provide opportunities of comparison in general and relative to certain reference groups (i.e., sex, age groups). In this study, we present such norm values (internationally comparable logit-transformed raw scores, percentiles, *z*-values and *T*-values) based on a representative German adult sample to facilitate the use of the SWEMWBS for research, professional practice and public health efforts. The provided normative values can be used as a benchmark for national and international comparisons both on the population and the individual level. In terms of more evidence-based mental health promotion measures, the provided norms can serve as a reference, for example when conducting impact analyses of targeted interventions for specific subgroups such as older people.

As part of a categorial approach we provide cut-off values for differentiating between low (≤ 23; logit-transformed 20.73), moderate (24 to 30; logit-transformed 21.54 to 27.03) and high mental well-being (≥ 32; logit-transformed 29.31). This enables a rather general classification and reporting of mental well-being at the population level in accordance with reporting customs for psychopathology markers (e.g., depressive symptoms with the PHQ-2) as used in the German MHS [25]. Consequently, public health efforts such as mental health promotion measures can be initiated, planned and evaluated continuously, evidence-based and precisely. For example, identifying at-risk groups with low mental well-being could facilitate targeted secondary prevention measures at the population level to improve mental well-being. In addition, comparison with standardized norms could help in setting up and evaluating smaller interventions and research, such as in clinical settings.

Mean mental well-being as well as the according German population norms were comparable to those of other European countries such as Iceland and Denmark and slightly higher than those estimated for the UK [26]. However, data from the Health Survey of England 2010–2013 [14] are already a little back in time, suggesting that mean well-being may have changed over the last 10 years. Also in line with the aforementioned studies is the present finding on relative similarity of SWEMWBS scores between women and men [14, 27], except for the youngest age group. In this study, the female young adults until about 35 years showed lower levels of mental well-being compared to males. The average mental well-being increased gradually with age until 89 years and was slightly lower in the group of the oldest-old (90–98 years). This corresponds with prior indications of higher mental well-being with increasing age [10, 26] and a decreasing trend in well-being observed for the oldest-old [28].

Since the WEMWBS and its short version SWEMWBS have been designed to be self-administered, reading the items aloud during the telephone interview may have increased the risk that participants respond more positively as compared to the situation of answering the items for themselves [6]. Future studies should take into account possible biases due to multimodal data collection procedures and conduct comparisons to test the validity of the SWEMWBS scale and the created norms measured across different collection modes. Moreover, persons with lower well-being might be less willing to participate, particularly in older age groups, which might have led to somewhat optimistic estimates.

### Electronic supplementary material

Below is the link to the electronic supplementary material.

Supplementary Material 1

## Abbreviations

MHS: Mental Health Surveillance
RKI: Robert Koch Institute
SWEMWBS: Short Warwick-Edinburgh Mental Well-Being Scale
UK: United Kingdom
WEMWBS: Warwick-Edinburgh Mental Well-Being Scale

## References

[1] Fusar-Poli, P., et al. (2020). What is good mental health? A scoping review. *European Neuropsychopharmacology*, *31*, 33–46. 31901337 10.1016/j.euroneuro.2019.12.105

[2] Iasiello, M., Van Agteren, J., & Muir-Cochrane, E. C. (2020). *Mental health and/or mental illness: A scoping review of the evidence and implications of the dual-continua model of mental health*. p. 1–45.

[3] World Health Organization, WHO. (2013). *Mental Health Action Plan 2013–2020*. p. 45.

[4] Choi, B. C. (2012). The past, present, and future of public health surveillance. *Scientifica (Cairo)*, *2012*, 875253. 10.6064/2012/875253 PMC3820481 24278752

[5] Thom, J., et al. (2021). Establishing a mental health surveillance in Germany: Development of a framework concept and indicator set. *Journal of Health Monitoring*, *6*(4), 34–63. 35146320 10.25646/8861 PMC8734140

[6] Stewart-Brown, S., & Janmohamed, K. (2008). *Warwick-Edinburgh mental well-being scale (WEMWBS). User guide. Version 1* (pp. 1–32). Warwick Medical School University of Warwick.

[7] Ryan, R. M., & Deci, E. L. (2001). On happiness and human potentials: A review of research on hedonic and eudaimonic well-being. *Annual Review of Psychology*, *52*, 141–166. 11148302 10.1146/annurev.psych.52.1.141

[8] Tennant, R., et al. (2007). The Warwick-Edinburgh mental well-being scale (WEMWBS): Development and UK validation. *Health and Quality of Life Outcomes*, *5*, 63. 18042300 10.1186/1477-7525-5-63 PMC2222612

[9] Stewart-Brown, S. L., et al. (2011). The Warwick-Edinburgh mental well-being scale (WEMWBS): A valid and reliable tool for measuring mental well-being in diverse populations and projects. *Journal of Epidemiology and Community Health*, *65*, A38–A39. 10.1136/jech.2011.143586.86

[10] Peitz, D., et al. (2023). *(Under review) validation of the Warwick Edinburgh mental well-being scale for the mental health surveillance (MHS) of German adults*. Health and Quality of Life Outcomes. 10.1186/s12955-024-02304-4 PMC11515111 39462382

[11] Stewart-Brown, S., et al. (2009). Internal construct validity of the Warwick-Edinburgh mental well-being scale (WEMWBS): A Rasch analysis using data from the Scottish health education population survey. *Health and Quality of Life Outcomes*, *7*, 15. 19228398 10.1186/1477-7525-7-15 PMC2669062

[12] Kobau, R., et al. (2011). Mental health promotion in public health: Perspectives and strategies from positive psychology. *American Journal of Public Health*, *101*(8), e1–9. 21680918 10.2105/AJPH.2010.300083 PMC3134513

[13] Vik, M. H., & Carlquist, E. (2018). Measuring subjective well-being for policy purposes: The example of well-being indicators in the WHO Health 2020 framework. *Scandinavian Journal of Public Health*, *46*(2), 279–286. 28830297 10.1177/1403494817724952

[14] Ng Fat, L., et al. (2017). Evaluating and establishing national norms for mental wellbeing using the short Warwick–Edinburgh mental well-being scale (SWEMWBS): Findings from the Health Survey for England. *Quality of Life Research*, *26*(5), 1129–1144. 27853963 10.1007/s11136-016-1454-8 PMC5376387

[15] Rosendahl Jensen, H. A., et al. (2022). The Danish health and wellbeing survey: Study design, response proportion and respondent characteristics. *Scandinavian Journal of Public Health*, *50*(7), 959–967. 34162289 10.1177/14034948211022429

[16] Shah, N., et al. (2021). Short Warwick-Edinburgh mental well-being scale (SWEMWBS): Performance in a clinical sample in relation to PHQ-9 and GAD-7. *Health and Quality of Life Outcomes*, *19*(1), 260. 34819104 10.1186/s12955-021-01882-x PMC8611866

[17] McKay, M. T., & Andretta, J. R. (2017). Evidence for the psychometric validity, internal consistency and measurement invariance of Warwick Edinburgh mental well-being scale scores in Scottish and Irish adolescents. *Psychiatry Research*, *255*, 382–386. 28666244 10.1016/j.psychres.2017.06.071

[18] Melendez-Torres, G. J., et al. (2019). Measurement invariance properties and external construct validity of the short Warwick-Edinburgh mental wellbeing scale in a large national sample of secondary school students in Wales. *Health and Quality of Life Outcomes*, *17*(1), 139. 31412878 10.1186/s12955-019-1204-z PMC6694652

[19] von der Heyde, C. (2013). *Das ADM-Stichprobensystem für Telefonbefragungen*. 10.25646/8559.

[20] Sand, M., & Gabler, S. (2019). Gewichtung Von (dual-Frame -) Telefonstichproben. In S. Häder, M. Häder, & P. Schmich (Eds.), *Telefonumfragen in Deutschland* (pp. 405–424). Springer.

[21] Allen, J., et al. (2021). German Health Update (GEDA 2019/2020-EHIS) - background and methodology. *Journal of Health Monitoring*, *6*(3), 66–79. 35146317 10.25646/8559 PMC8734110

[22] Lang, G., & Bachinger, A. (2017). Validation of the German Warwick-Edinburgh mental well-being scale (WEMWBS) in a community-based sample of adults in Austria: A bi-factor modelling approach. *Journal of Public Health (Oxford, England)*, *25*, 135–146. 10.1007/s10389-016-0778-8

[23] R Core Team. (2021). *A language and environment for statistical computing*. Available from: https://www.r-project.org/.

[24] Lenhard, A., Lenhard, W., & Gary, S. (2018). *cNORM - Generating continuous test norms*. Retrieved from: https://www.psychometrica.de/cNorm_en.html. Psychometrica.

[25] Mauz, E., et al. (2023). Time trends in mental health indicators in Germany’s adult population before and during the COVID-19 pandemic. *Front Public Health*, *11*, 1065938. 36908429 10.3389/fpubh.2023.1065938 PMC9995751

[26] Koushede, V., et al. (2019). Measuring mental well-being in Denmark: Validation of the original and short version of the Warwick-Edinburgh mental well-being scale (WEMWBS and SWEMWBS) and cross-cultural comparison across four European settings. *Psychiatry Research*, *271*, 502–509. 30551082 10.1016/j.psychres.2018.12.003

[27] Haver, A., et al. (2015). Measuring mental well-being: A validation of the short Warwick-Edinburgh mental well-being scale in Norwegian and Swedish. *Scandinavian Journal of Public Health*, *43*(7), 721–727. 26041133 10.1177/1403494815588862

[28] Vanhoutte, B., & Nazroo, J. (2014). Cognitive, affective and eudemonic well-being in later life: Measurement equivalence over gender and life stage. *Sociol Responsibility Online*, 19(2). 10.5153/sro.3241 PMC4943521 27429579

