# Supplementary material for: German adult population norm values of the short Warwick Edinburgh mental well-being scale (SWEMWBS)
Source: Qual Life Res. 2024 Jun 5;33(8):2145–50. doi: 10.1007/s11136-024-03695-z (PMC11286698; doi:10.1007/s11136-024-03695-z)
Supplement: Supplementary file 1 — Supplementary Material 1 [file 11136_2024_3695_MOESM1_ESM.docx]

| **Table S1**  Smoothed SWEMWBS percentiles, z-values, T-values, raw scores and logit-transformed scores, based on *N* = 5,606 German adults at different ages separated into 5-year sections | | | | | |
| --- | --- | --- | --- | --- | --- |
| **Percentile** | ***T*-value** | ***z*-value** | **Age** | **Raw score** | **Logit-transformed** |
| 2.5 | 30.40 | -1.96 | 18-19 | 17.5 | 17.43 |
|  |  |  | 20-24 | 17.7 | 17.43 |
|  |  |  | 25-29 | 17.9 | 17.43 |
|  |  |  | 30-34 | 18.1 | 17.43 |
|  |  |  | 35-39 | 18.2 | 17.43 |
|  |  |  | 40-44 | 18.4 | 17.43 |
|  |  |  | 45-49 | 18.5 | 17.98 |
|  |  |  | 50-54 | 18.6 | 17.98 |
|  |  |  | 55-59 | 18.7 | 17.98 |
|  |  |  | 60-64 | 18.8 | 17.98 |
|  |  |  | 65-69 | 18.8 | 17.98 |
|  |  |  | 70-74 | 18.9 | 17.98 |
|  |  |  | 75-79 | 18.9 | 17.98 |
|  |  |  | 80-84 | 18.9 | 17.98 |
|  |  |  | 85-89 | 18.9 | 17.98 |
|  |  |  | 90-98 | 18.9 | 17.98 |
| 15 | 39.64 | -1.03 | 18-19 | 22.1 | 19.98 |
|  |  |  | 20-24 | 22.3 | 19.98 |
|  |  |  | 25-29 | 22.6 | 20.73 |
|  |  |  | 30-34 | 22.8 | 20.73 |
|  |  |  | 35-39 | 23.0 | 20.73 |
|  |  |  | 40-44 | 23.2 | 20.73 |
|  |  |  | 45-49 | 23.3 | 20.73 |
|  |  |  | 50-54 | 23.5 | 21.54 |
|  |  |  | 55-59 | 23.6 | 21.54 |
|  |  |  | 60-64 | 23.7 | 21.54 |
|  |  |  | 65-69 | 23.8 | 21.54 |
|  |  |  | 70-74 | 23.8 | 21.54 |
|  |  |  | 75-79 | 23.9 | 21.54 |
|  |  |  | 80-84 | 23.9 | 21.54 |
|  |  |  | 85-89 | 23.9 | 21.54 |
|  |  |  | 90-98 | 23.9 | 21.54 |
| 25 | 43.26 | -0.67 | 18-19 | 23.7 | 21.54 |
|  |  |  | 20-24 | 23.9 | 21.54 |
|  |  |  | 25-29 | 24.2 | 21.54 |
|  |  |  | 30-34 | 24.4 | 21.54 |
|  |  |  | 35-39 | 24.7 | 22.35 |
|  |  |  | 40-44 | 24.9 | 22.35 |
|  |  |  | 45-49 | 25.0 | 22.35 |
|  |  |  | 50-54 | 25.2 | 22.35 |
|  |  |  | 55-59 | 25.3 | 22.35 |
|  |  |  | 60-64 | 25.4 | 22.35 |
|  |  |  | 65-69 | 25.5 | 23.21 |
|  |  |  | 70-74 | 25.6 | 23.21 |
|  |  |  | 75-79 | 25.6 | 23.21 |
|  |  |  | 80-84 | 25.7 | 23.21 |
|  |  |  | 85-89 | 25.7 | 23.21 |
|  |  |  | 90-98 | 25.6 | 23.21 |
| 50 | 50.00 | 0.00 | 18-19 | 26.2 | 23.21 |
|  |  |  | 20-24 | 26.6 | 24.11 |
|  |  |  | 25-29 | 26.9 | 24.11 |
|  |  |  | 30-34 | 27.2 | 24.11 |
|  |  |  | 35-39 | 27.4 | 24.11 |
|  |  |  | 40-44 | 27.6 | 25.03 |
|  |  |  | 45-49 | 27.8 | 25.03 |
|  |  |  | 50-54 | 28.0 | 25.03 |
|  |  |  | 55-59 | 28.2 | 25.03 |
|  |  |  | 60-64 | 28.3 | 25.03 |
|  |  |  | 65-69 | 28.4 | 25.03 |
|  |  |  | 70-74 | 28.5 | 26.02 |
|  |  |  | 75-79 | 28.5 | 26.02 |
|  |  |  | 80-84 | 28.6 | 26.02 |
|  |  |  | 85-89 | 28.6 | 26.02 |
|  |  |  | 90-98 | 28.5 | 26.02 |
| 75 | 56.74 | 0.67 | 18-19 | 28.3 | 25.03 |
|  |  |  | 20-24 | 28.7 | 26.02 |
|  |  |  | 25-29 | 29.1 | 26.02 |
|  |  |  | 30-34 | 29.4 | 26.02 |
|  |  |  | 35-39 | 29.7 | 27.03 |
|  |  |  | 40-44 | 29.9 | 27.03 |
|  |  |  | 45-49 | 30.2 | 27.03 |
|  |  |  | 50-54 | 30.4 | 27.03 |
|  |  |  | 55-59 | 30.5 | 28.13 |
|  |  |  | 60-64 | 30.7 | 28.13 |
|  |  |  | 65-69 | 30.8 | 28.13 |
|  |  |  | 70-74 | 30.9 | 28.13 |
|  |  |  | 75-79 | 30.9 | 28.13 |
|  |  |  | 80-84 | 31.0 | 28.13 |
|  |  |  | 85-89 | 31.0 | 28.13 |
|  |  |  | 90-98 | 31.0 | 28.13 |
| 85 | 60.36 | 1.04 | 18-19 | 29.3 | 26.02 |
|  |  |  | 20-24 | 29.7 | 27.03 |
|  |  |  | 25-29 | 30.1 | 27.03 |
|  |  |  | 30-34 | 30.4 | 27.03 |
|  |  |  | 35-39 | 30.7 | 28.13 |
|  |  |  | 40-44 | 31.0 | 28.13 |
|  |  |  | 45-49 | 31.2 | 28.13 |
|  |  |  | 50-54 | 31.5 | 29.31 |
|  |  |  | 55-59 | 31.6 | 29.31 |
|  |  |  | 60-64 | 31.8 | 29.31 |
|  |  |  | 65-69 | 31.9 | 29.31 |
|  |  |  | 70-74 | 32.0 | 29.31 |
|  |  |  | 75-79 | 32.1 | 29.31 |
|  |  |  | 80-84 | 32.1 | 29.31 |
|  |  |  | 85-89 | 32.1 | 29.31 |
|  |  |  | 90-98 | 32.1 | 29.31 |
| 97.5 | 69.60 | 1.96 | 18-19 | 31.6 | 29.31 |
|  |  |  | 20-24 | 32.1 | 29.31 |
|  |  |  | 25-29 | 32.5 | 30.70 |
|  |  |  | 30-34 | 32.9 | 30.70 |
|  |  |  | 35-39 | 33.3 | 30.70 |
|  |  |  | 40-44 | 33.6 | 32.55 |
|  |  |  | 45-49 | 33.9 | 32.55 |
|  |  |  | 50-54 | 34.1 | 32.55 |
|  |  |  | 55-59 | 34.3 | 32.55 |
|  |  |  | 60-64 | 34.5 | 35.00 |
|  |  |  | 65-69 | 34.6 | 35.00 |
|  |  |  | 70-74 | 34.8 | 35.00 |
|  |  |  | 75-79 | 34.8 | 35.00 |
|  |  |  | 80-84 | 34.9 | 35.00 |
|  |  |  | 85-89 | 34.9 | 35.00 |
|  |  |  | 90-98 | 34.8 | 35.00 |
| *Notes:* The smoothed values represent the model predictions for the midpoint of each age group, e.g. the prediction for age 32.5 is shown for the age group 30-34 years. For the highest age group, the prediction for 92.5 years is shown. The allocation of logit-transformed values to the (rounded) raw scores was based on Stewart-Brown and colleagues [11]. | | | | | |

| **Table S2**  Smoothed SWEMWBS percentiles, z-values, T-values, raw scores and logit-transformed scores, based on *N* = 5,606 German adults stratified by sex and at different ages separated into 5-year sections | | | | | | | |
| --- | --- | --- | --- | --- | --- | --- | --- |
|  |  |  |  | ***Male*** | | ***Female*** | |
| **Percentile** | ***T*-value** | ***z*-value** | **Age** | **Raw score** | **Logit-transformed** | **Raw score** | **Logit-transformed** |
| 2.5 | 30.40 | -1.96 | 18-19 | 18.1 | 17.43 | 16.2 | 16.36 |
|  |  |  | 20-24 | 18.1 | 17.43 | 16.9 | 16.88 |
|  |  |  | 25-29 | 18.2 | 17.43 | 17.5 | 17.43 |
|  |  |  | 30-34 | 18.2 | 17.43 | 18.0 | 17.43 |
|  |  |  | 35-39 | 18.2 | 17.43 | 18.4 | 17.43 |
|  |  |  | 40-44 | 18.2 | 17.43 | 18.8 | 17.98 |
|  |  |  | 45-49 | 18.2 | 17.43 | 19.2 | 17.98 |
|  |  |  | 50-54 | 18.3 | 17.43 | 19.4 | 17.98 |
|  |  |  | 55-59 | 18.3 | 17.43 | 19.6 | 18.59 |
|  |  |  | 60-64 | 18.3 | 17.43 | 19.8 | 18.59 |
|  |  |  | 65-69 | 18.4 | 17.43 | 19.8 | 18.59 |
|  |  |  | 70-74 | 18.4 | 17.43 | 19.9 | 18.59 |
|  |  |  | 75-79 | 18.5 | 17.98 | 19.8 | 18.59 |
|  |  |  | 80-84 | 18.5 | 17.98 | 19.7 | 18.59 |
|  |  |  | 85-89 | 18.6 | 17.98 | 19.5 | 18.59 |
|  |  |  | 90-98 | 18.6 | 17.98 | 19.3 | 17.98 |
| 15 | 39.64 | -1.03 | 18-19 | 22.9 | 20.73 | 20.8 | 19.25 |
|  |  |  | 20-24 | 22.9 | 20.73 | 21.4 | 19.25 |
|  |  |  | 25-29 | 23.0 | 20.73 | 22.0 | 19.98 |
|  |  |  | 30-34 | 23.0 | 20.73 | 22.5 | 20.73 |
|  |  |  | 35-39 | 23.0 | 20.73 | 23.0 | 20.73 |
|  |  |  | 40-44 | 23.1 | 20.73 | 23.4 | 20.73 |
|  |  |  | 45-49 | 23.2 | 20.73 | 23.7 | 21.54 |
|  |  |  | 50-54 | 23.2 | 20.73 | 24.0 | 21.54 |
|  |  |  | 55-59 | 23.3 | 20.73 | 24.2 | 21.54 |
|  |  |  | 60-64 | 23.4 | 20.73 | 24.3 | 21.54 |
|  |  |  | 65-69 | 23.4 | 20.73 | 24.4 | 21.54 |
|  |  |  | 70-74 | 23.5 | 21.54 | 24.4 | 21.54 |
|  |  |  | 75-79 | 23.6 | 21.54 | 24.4 | 21.54 |
|  |  |  | 80-84 | 23.7 | 21.54 | 24.2 | 21.54 |
|  |  |  | 85-89 | 23.8 | 21.54 | 24.1 | 21.54 |
|  |  |  | 90-98 | 23.9 | 21.54 | 23.8 | 21.54 |
| 25 | 43.26 | -0.67 | 18-19 | 24.6 | 22.35 | 22.4 | 20.73 |
|  |  |  | 20-24 | 24.6 | 22.35 | 23.0 | 20.73 |
|  |  |  | 25-29 | 24.6 | 22.35 | 23.6 | 21.54 |
|  |  |  | 30-34 | 24.7 | 22.35 | 24.1 | 21.54 |
|  |  |  | 35-39 | 24.8 | 22.35 | 24.6 | 22.35 |
|  |  |  | 40-44 | 24.8 | 22.35 | 25.0 | 22.35 |
|  |  |  | 45-49 | 24.9 | 22.35 | 25.3 | 22.35 |
|  |  |  | 50-54 | 25.0 | 22.35 | 25.6 | 23.21 |
|  |  |  | 55-59 | 25.1 | 22.35 | 25.8 | 23.21 |
|  |  |  | 60-64 | 25.1 | 22.35 | 25.9 | 23.21 |
|  |  |  | 65-69 | 25.2 | 22.35 | 26.0 | 23.21 |
|  |  |  | 70-74 | 25.3 | 22.35 | 26.0 | 23.21 |
|  |  |  | 75-79 | 25.4 | 22.35 | 26.0 | 23.21 |
|  |  |  | 80-84 | 25.5 | 23.21 | 25.9 | 23.21 |
|  |  |  | 85-89 | 25.6 | 23.21 | 25.7 | 23.21 |
|  |  |  | 90-98 | 25.7 | 23.21 | 25.4 | 22.35 |
| 50 | 50.0 | 0.0 | 18-19 | 27.3 | 24.11 | 25.0 | 22.35 |
|  |  |  | 20-24 | 27.3 | 24.11 | 25.7 | 23.21 |
|  |  |  | 25-29 | 27.4 | 24.11 | 26.3 | 23.21 |
|  |  |  | 30-34 | 27.5 | 25.03 | 26.8 | 24.11 |
|  |  |  | 35-39 | 27.6 | 25.03 | 27.2 | 24.11 |
|  |  |  | 40-44 | 27.7 | 25.03 | 27.6 | 25.03 |
|  |  |  | 45-49 | 27.8 | 25.03 | 28.0 | 25.03 |
|  |  |  | 50-54 | 27.9 | 25.03 | 28.2 | 25.03 |
|  |  |  | 55-59 | 28.0 | 25.03 | 28.4 | 25.03 |
|  |  |  | 60-64 | 28.1 | 25.03 | 28.6 | 26.02 |
|  |  |  | 65-69 | 28.2 | 25.03 | 28.7 | 26.02 |
|  |  |  | 70-74 | 28.4 | 25.03 | 28.7 | 26.02 |
|  |  |  | 75-79 | 28.5 | 26.02 | 28.6 | 26.02 |
|  |  |  | 80-84 | 28.6 | 26.02 | 28.5 | 26.02 |
|  |  |  | 85-89 | 28.8 | 26.02 | 28.3 | 25.03 |
|  |  |  | 90-98 | 28.9 | 26.02 | 28.1 | 25.03 |
| 75 | 56.74 | 0.67 | 18-19 | 29.5 | 27.03 | 27.3 | 24.11 |
|  |  |  | 20-24 | 29.6 | 27.03 | 27.9 | 25.03 |
|  |  |  | 25-29 | 29.7 | 27.03 | 28.5 | 26.02 |
|  |  |  | 30-34 | 29.8 | 27.03 | 29.0 | 26.02 |
|  |  |  | 35-39 | 29.9 | 27.03 | 29.5 | 27.03 |
|  |  |  | 40-44 | 30.0 | 27.03 | 29.9 | 27.03 |
|  |  |  | 45-49 | 30.1 | 27.03 | 30.9 | 28.13 |
|  |  |  | 50-54 | 30.3 | 27.03 | 30.4 | 27.03 |
|  |  |  | 55-59 | 30.4 | 27.03 | 30.7 | 28.13 |
|  |  |  | 60-64 | 30.6 | 28.13 | 30.8 | 28.13 |
|  |  |  | 65-69 | 30.7 | 28.13 | 30.9 | 28.13 |
|  |  |  | 70-74 | 30.9 | 28.13 | 30.9 | 28.13 |
|  |  |  | 75-79 | 31.0 | 28.13 | 30.8 | 28.13 |
|  |  |  | 80-84 | 31.2 | 28.13 | 30.7 | 28.13 |
|  |  |  | 85-89 | 31.3 | 28.13 | 30.6 | 28.13 |
|  |  |  | 90-98 | 31.5 | 29.31 | 30.3 | 27.03 |
| 85 | 60.36 | 1.04 | 18-19 | 30.5 | 28.13 | 28.3 | 25.03 |
|  |  |  | 20-24 | 30.6 | 28.13 | 29.0 | 26.02 |
|  |  |  | 25-29 | 30.7 | 28.13 | 29.5 | 27.03 |
|  |  |  | 30-34 | 30.8 | 28.13 | 30.1 | 27.03 |
|  |  |  | 35-39 | 31.0 | 28.13 | 30.5 | 28.13 |
|  |  |  | 40-44 | 31.1 | 28.13 | 30.9 | 28.13 |
|  |  |  | 45-49 | 31.3 | 28.13 | 31.2 | 28.13 |
|  |  |  | 50-54 | 31.4 | 28.13 | 31.5 | 29.31 |
|  |  |  | 55-59 | 31.6 | 29.31 | 31.7 | 29.31 |
|  |  |  | 60-64 | 31.7 | 29.31 | 31.9 | 29.31 |
|  |  |  | 65-69 | 31.9 | 29.31 | 31.9 | 29.31 |
|  |  |  | 70-74 | 32.1 | 29.31 | 31.9 | 29.31 |
|  |  |  | 75-79 | 32.2 | 29.31 | 31.9 | 29.31 |
|  |  |  | 80-84 | 32.3 | 29.31 | 31.8 | 29.31 |
|  |  |  | 85-89 | 32.5 | 30.70 | 31.6 | 29.31 |
|  |  |  | 90-98 | 32.6 | 30.70 | 31.4 | 28.13 |
| 97.5 | 69.60 | 1.96 | 18-19 | 32.9 | 30.70 | 30.9 | 28.13 |
|  |  |  | 20-24 | 33.0 | 30.70 | 31.6 | 29.31 |
|  |  |  | 25-29 | 33.2 | 30.70 | 32.2 | 29.31 |
|  |  |  | 30-34 | 33.3 | 30.70 | 32.7 | 30.70 |
|  |  |  | 35-39 | 33.5 | 32.55 | 33.1 | 30.70 |
|  |  |  | 40-44 | 33.7 | 32.55 | 33.5 | 32.55 |
|  |  |  | 45-49 | 33.9 | 32.55 | 33.8 | 32.55 |
|  |  |  | 50-54 | 34.1 | 32.55 | 34.1 | 32.55 |
|  |  |  | 55-59 | 34.3 | 32.55 | 34.3 | 32.55 |
|  |  |  | 60-64 | 34.4 | 32.55 | 34.5 | 35.00 |
|  |  |  | 65-69 | 34.6 | 35.00 | 34.5 | 35.00 |
|  |  |  | 70-74 | 34.7 | 35.00 | 34.6 | 35.00 |
|  |  |  | 75-79 | 34.8 | 35.00 | 34.5 | 35.00 |
|  |  |  | 80-84 | 34.9 | 35.00 | 34.4 | 32.55 |
|  |  |  | 85-89 | 34.9 | 35.00 | 34.2 | 32.55 |
|  |  |  | 90-98 | 35.0 | 35.00 | 34.0 | 32.55 |
| *Notes:* The smoothed values represent the model predictions for the midpoint of each age group, e.g. the prediction for age 32.5 is shown for the age group 30-34 years. For the highest age group, the prediction for 92.5 years is shown. The allocation of logit-transformed values to the (rounded) raw scores was based on Stewart-Brown and colleagues [11]. | | | | | | | |


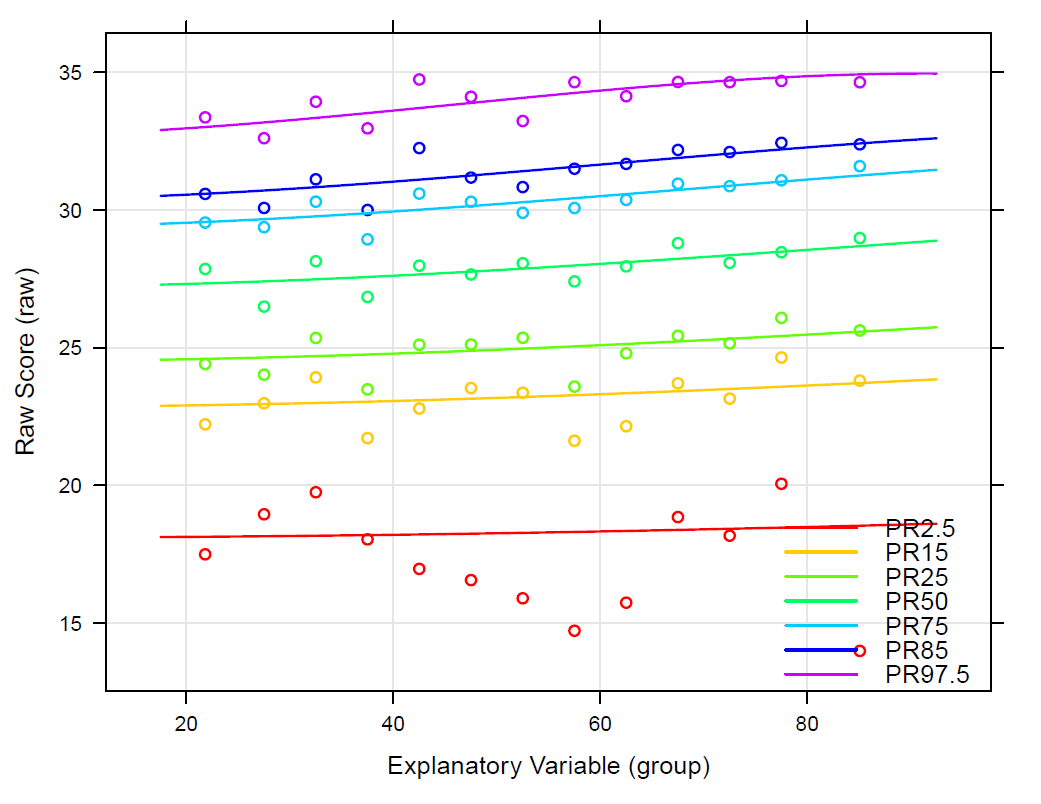


**Figure S1.** Smoothed norm curves against percentiles by age for male participants


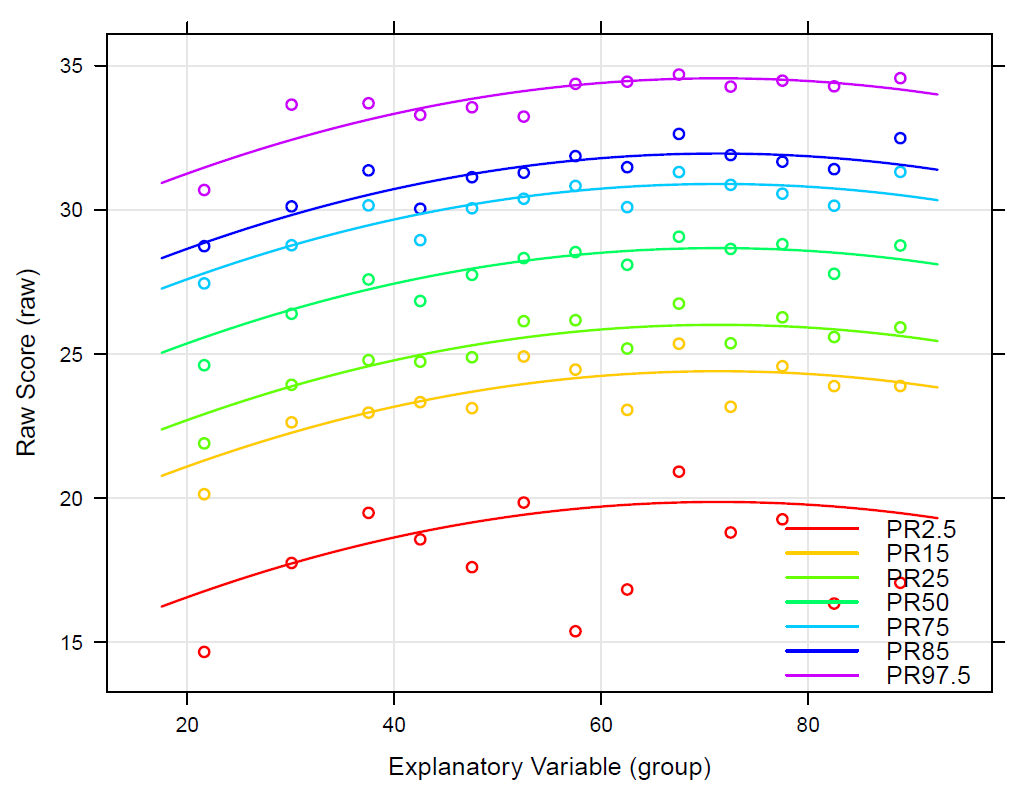
**Figure S2.** Smoothed norm curves against percentiles by age for female participants
